# Distinct effects of Q925 mutation on intracellular and extracellular Na^+^ and K^+^ binding to the Na^+^, K^+^-ATPase

**DOI:** 10.1038/s41598-019-50009-2

**Published:** 2019-09-16

**Authors:** Hang N. Nielsen, Kerri Spontarelli, Rikke Holm, Jens Peter Andersen, Anja P. Einholm, Pablo Artigas, Bente Vilsen

**Affiliations:** 1 Department of Biomedicine, Aarhus University, DK-8000 Aarhus C, Denmark; 2 Department of Cell Physiology and Molecular Biophysics, Center for Membrane Protein Research, Texas Tech University Health Sciences Center, Lubbock, TX 79430 USA

**Keywords:** Permeation and transport, Enzyme mechanisms

## Abstract

Three Na^+^ sites are defined in the Na^+^-bound crystal structure of Na^+^, K^+^-ATPase. Sites I and II overlap with two K^+^ sites in the K^+^-bound structure, whereas site III is unique and Na^+^ specific. A glutamine in transmembrane helix M8 (Q925) appears from the crystal structures to coordinate Na^+^ at site III, but does not contribute to K^+^ coordination at sites I and II. Here we address the functional role of Q925 in the various conformational states of Na^+^, K^+^-ATPase by examining the mutants Q925A/G/E/N/L/I/Y. We characterized these mutants both enzymatically and electrophysiologically, thereby revealing their Na^+^ and K^+^ binding properties. Remarkably, Q925 substitutions had minor effects on Na^+^ binding from the intracellular side of the membrane – in fact, mutations Q925A and Q925G increased the apparent Na^+^ affinity – but caused dramatic reductions of the binding of K^+^ as well as Na^+^ from the extracellular side of the membrane. These results provide insight into the changes taking place in the Na^+^-binding sites, when they are transformed from intracellular- to extracellular-facing orientation in relation to the ion translocation process, and demonstrate the interaction between sites III and I and a possible gating function of Q925 in the release of Na^+^ at the extracellular side.

## Introduction

**Figure 1:**
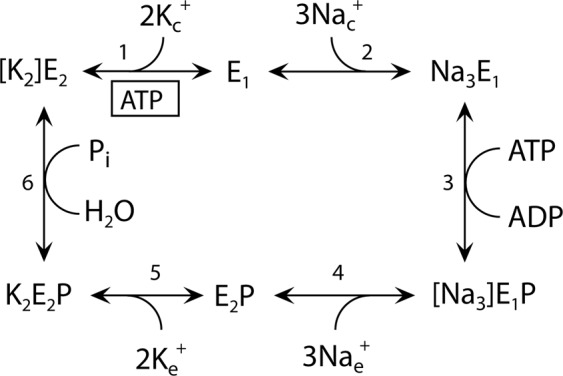
Na^+^, K^+^-ATPase reaction cycle. E_1_ and E_2_ represent the main conformational states of the enzyme. P indicates phosphorylated states. E_1_P and E_2_P are the respective ADP-sensitive and ADP-insensitive (K^+^ sensitive) phosphoenzyme intermediates^2^. Occluded Na^+^ and K^+^ ions^3^ are shown in brackets. Free ions are labelled c and e for cytoplasmic and extracellular side, respectively. Boxed ATP indicates ATP bound in a non-phosphorylating mode, enhancing the rate of K^+^ deocclusion, and accompanying E_2_-E_1_ conformational change^2^. The partial reactions of the cycle are numbered 1–6.

The Na^+^, K^+^-ATPase is found in all animal cells, where it creates vital Na^+^ and K^+^ gradients across the cell membrane by exchanging 3 cytoplasmic Na^+^ for 2 extracellular K^+^ per ATP hydrolysed, in a consecutive transport mode where Na^+^ is translocated before K^+ ^^1–4^. During the transport cycle, the Na^+^, K^+^-ATPase alternates between two major conformational states, E_1_ and E_2_, with preferential binding of Na^+^ and K^+^, respectively (Fig. 1). The Na^+^, K^+^-ATPase is composed of α-, β-, and γ-subunits, of which the α-subunit contains the binding sites for ATP and the transported ions, as well as sites binding the inhibitors ouabain and oligomycin. The transmembrane part of the α-subunit consists of 10 transmembrane spanning helices M1-M10, of which M4, M5, M6, and M8 host residues involved in the binding of Na^+^ and K^+ ^^5–14^. The two K^+^ sites overlap with two of the three Na^+^ sites, denoted sites I and II, whereas Na^+^ site III seems to be unique and Na^+^ specific. The three intracellular Na^+^ ions appear to bind in a sequential and cooperative manner^14,15^. The glutamine of M8, Q925 (rat α_1_ numbering, as used throughout this article), has been suggested to be essential for Na^+^, K^+^-ATPase activity^11^, and is one of the residues within coordination distance of the Na^+^ ion at site III in the Na^+^-bound crystal structure^14^. Furthermore, mutation to proline of the equivalent residue (Q927) in the human α2-isoform causes hemiplegic migraine^16^, highlighting its importance. However, no functional evidence has so far demonstrated any role of Q925 in Na^+^ or K^+^ binding. The static crystal structure lacks information on which residues are critical to ion binding during the dynamic process where the Na^+^ sites switch between intracellular- and extracellular-facing orientations in relation to the ion translocation process. In the present study, we have addressed the specific functional role of Q925 in the various conformational states of the Na^+^, K^+^-ATPase by examining the mutants Q925A/G/E/N/L/I/Y. We have characterized these mutants enzymatically, particularly with respect to their sided interaction with Na^+^ and K^+^. Study of the Na^+^ dependence of phosphorylation from ATP provided information on Na^+^ interaction at the intracellular-facing sites. To evaluate the interaction with Na^+^ and K^+^ at the extracellular-facing sites, we conducted electrophysiological experiments. Unexpectedly, the findings show that Q925 contributes little to Na^+^ binding from the intracellular side – in fact, mutations Q925A and Q925G were compatible with Na^+^ and K^+^ transport and ATPase activity and increased the apparent Na^+^ affinity, despite removing an oxygen presumed to participate in Na^+^-coordination. On the other hand, Q925 plays a crucial role in the binding of Na^+^ and K^+^ from the extracellular side of the membrane.

## Results

### Stable and transient expression of Q925 mutants

For enzymatic studies, the mutations corresponding to Q925A/G/E/N/L/I/Y were introduced in the cDNA encoding the relatively ouabain-insensitive rat α_1_ Na^+^, K^+^-ATPase, and these constructs were expressed by transfection of mammalian cells (COS-1). Because Na^+^, K^+^-ATPase is endogenous to all mammalian cells, a special strategy is required to characterize exogenously expressed Na^+^, K^+^-ATPase without interference from the endogenous COS-1 cell enzyme^5,17,18^. The >100-fold higher sensitivity of the endogenous COS-1 cell Na^+^, K^+^-ATPase to the inhibitor ouabain, as compared with the rat α_1_ Na^+^, K^+^-ATPase, allows the use of ouabain to specifically inhibit the endogenous enzyme without affecting the exogenous rat α_1_ Na^+^, K^+^-ATPase. Thus, if the exogenous enzyme actively transports Na^+^ and K^+^, stable cell lines expressing it can be isolated under ouabain selection pressure, using ouabain to inhibit the endogenous ouabain-sensitive Na^+^, K^+^-ATPase of the cells. However, a transport-inactive exogenous enzyme would be unable to support cell growth, and the cells would die in the presence of ouabain inhibiting the endogenous Na^+^, K^+^-ATPase, because active Na^+^ and K^+^ transport is mandatory for cell viability. Like the wild type rat α_1_ Na^+^, K^+^-ATPase, the mutants Q925A and Q925G sustained cell viability in the presence of ouabain, indicating retained transport activity. On the other hand, the remaining five mutants Q925E/N/L/I/Y were unable to support cell growth, indicating that they were inactive under physiological conditions, precluding isolation of stable cell lines. Therefore, we applied a transient expression strategy to study the latter mutants. The ratio between the exogenously expressed enzyme and the endogenous COS-1 cell enzyme background obtained by transient expression was lower than that obtained by stable expression, but it was improved by addition of siRNA that specifically knocks down expression of the endogenous enzyme (see Methods). In addition, for both the stably and the transiently expressed mutants, functional assays were carried out in the presence of ouabain at a concentration that completely silences the endogenous COS-1 cell enzyme. To evaluate whether the functional characteristics determined depended on the expression strategy, the transport-active Q925A mutant and the wild type were expressed both stably and transiently using the siRNA methodology.

### Apparent Na^+^ affinity of Q925 mutants

**Figure 2:**
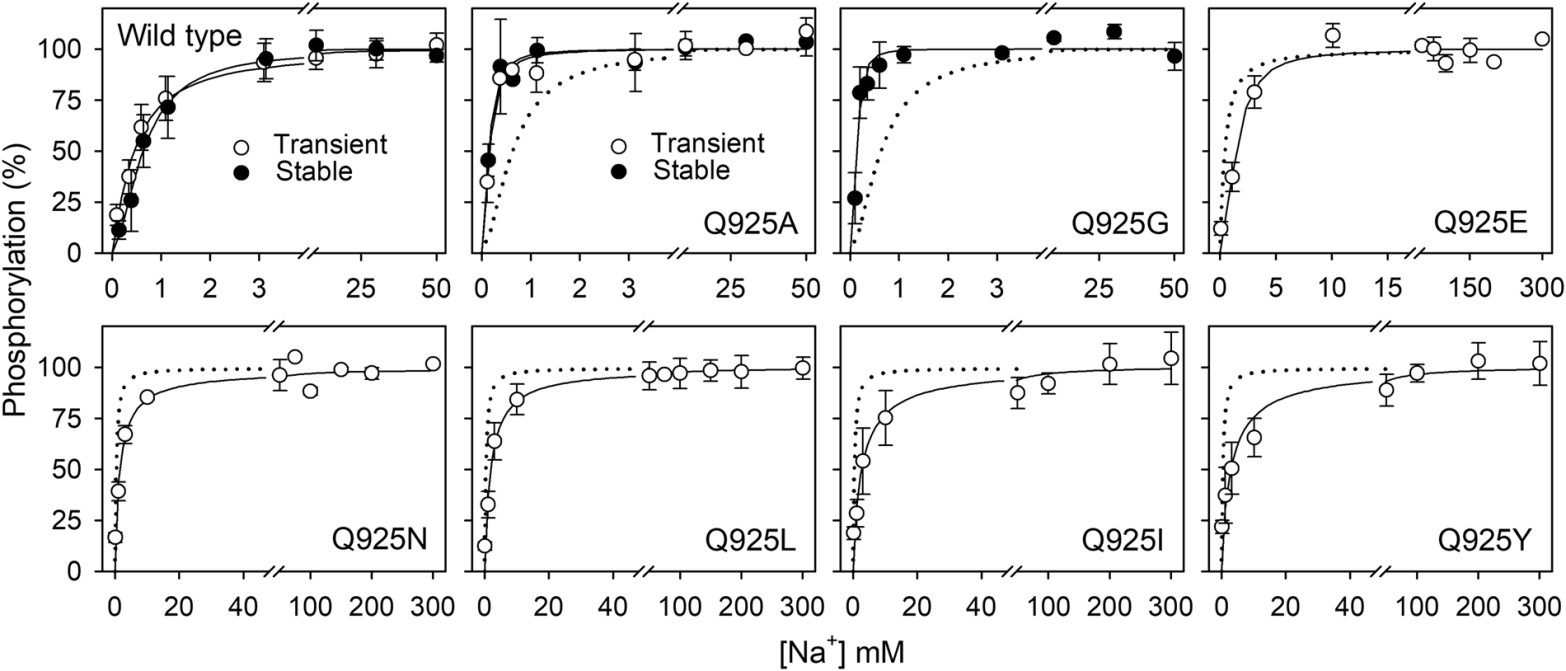
Na^+^ dependence of phosphorylation. Phosphorylation was carried out for 10 s at 0 °C with 2 μM [γ-^32^P]ATP in 20 mM Tris (pH 7.5), 3 mM MgCl_2_, 1 mM EGTA, 20 μg oligomycin/ml (to prevent dephosphorylation), ouabain to inhibit the endogenous COS-1 cell Na^+^, K^+^-ATPase (10 μM for stably expressed enzyme and 100 μM for transiently expressed enzyme), and various concentrations of NaCl (and NMG^+^ to maintain the same ionic strength at all NaCl concentrations). For further details, see Methods. Symbols with error bars represent mean ± s.d. Open and closed symbols represent transiently and stably expressed enzyme, respectively. Each line represents the best fit of a Hill function (Eq. 1 of Methods). Extracted K_0.5_ values are listed in Table 1 with statistical information. Dotted lines reproduce the corresponding wild type for direct comparison in the same panel (only the stably expressed wild type is shown in the panel with Q925A).

All the enzymatic studies described below were performed with isolated plasma membranes made leaky by addition of the pore-forming compound alamethicin to allow access of the added Na^+^, K^+^, and ATP to the enzyme from both sides of the membrane. The apparent affinity for Na^+^ binding at the intracellular-facing sites was determined by studying the Na^+^ dependence of phosphorylation from [γ-^32^P]ATP (Fig. 2). The phosphorylation of wild type Na^+^, K^+^-ATPase requires the binding of all 3 Na^+^ ions (Fig. 1, reactions 2 and 3). The assay was carried out in the presence of oligomycin, which hinders dephosphorylation, as it stabilizes the enzyme in the occluded [Na_3_]E_1_P form^3^. Both the transport-active Q925A/G mutants and the transport-inactive Q925E/N/L/I/Y mutants were able to form a phosphoenzyme in the presence of ATP. The apparent affinity for Na^+^ of the wild type and the Q925A mutant was independent of the method of transfection (compare open and closed circles of Fig. 2, see also Table 1), thus validating the transient transfection/siRNA method. Importantly, the Q925A and Q925G mutants exhibited 2- to 3-fold higher apparent Na^+^ affinity (K_0.5_ reduced) than the wild type, which is remarkable, considering that the position of Q925 in the crystal structure qualifies it as a ligand of Na^+^ in site III. By contrast, the apparent Na^+^ affinity was reduced by substituents with larger side chains. The most bulky hydrophobic and aromatic residues (leucine, isoleucine, and tyrosine) yielded a 4- to 6-fold reduction, whereas asparagine and glutamate induced a 3-fold reduction in Na^+^ affinity (Fig. 2 and Table 1).

**Table 1:** Functional analysis by phosphorylation measurements.

	Na^+^ activation of phosphorylation	K^+^ inhibition of phosphorylation	E_2_P
	K_0.5_ (Na^+^)	IC_50_ (K^+^)	
	μM	mM	(%)
α1 Wt (stable)	502 ± 96 n = 13	~0.05 n = 7	61 ± 9 n = 10
α1 Wt (transient)	473 ± 91 n = 20	~0.05 n = 18	61 ± 10 n = 12
Q925A stable	174 ± 36 (2.9 ×↓) n = 6	~400 ×↑ n = 5	65 ± 13 n = 3
Q925A transient	253 ± 116 (1.9 ×↓) n = 6	~400 ×↑ n = 3	59 ± 7 n = 3
Q925G stable	184 ± 51 (2.7 ×↓) n = 4	~400 ×↑ n = 4	54 ± 8 n = 2
Q925E	1,461 ± 245 (3.1 ×↑) n = 4¤	~1,000 ×↑ n = 6	93 ± 3 n = 2
Q925N	1,571 ± 219 (3.3 ×↑) n = 3	~400 ×↑ n = 8	100 ± 0.3 n = 3
Q925L	1,988 ± 660 (4.2 ×↑) n = 10	> 1,000 ×↑ n = 11	73 ± 10 n = 3
Q925I	3,047 ± 1650 (6.4 ×↑) n = 2	> 1,000 ×↑ n = 4	72 ± 4 n = 2
Q925Y	2,986 ± 1232 (6.3 ×↑) n = 5	> 1,000 ×↑ n = 5	61 ± 9 n = 2

**Figure 3:**
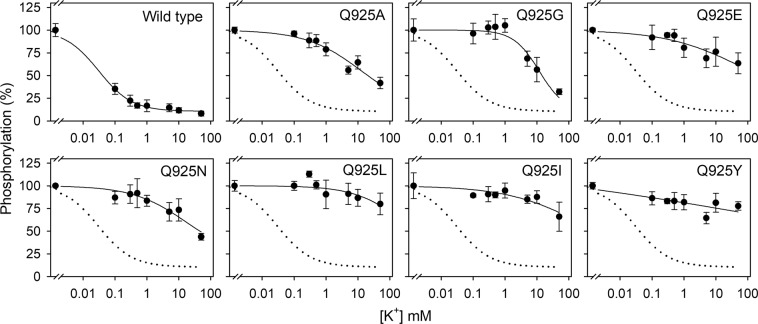
K^+^ inhibition of phosphorylation. Phosphorylation with [γ-^32^P]ATP was carried out for 10 s at 0 °C with 2 μM [γ-^32^P]ATP in 20 mM Tris (pH 7.5), 50 mM NaCl, 3 mM MgCl_2_, 1 mM EGTA, ouabain to inhibit the endogenous COS-1 cell Na^+^, K^+^-ATPase, and various concentrations of KCl (with choline chloride to maintain a constant ionic strength). In these experiments, oligomycin was absent to allow dephosphorylation. For further details, see Methods. Symbols with error bars represent mean ± s.d. Each line represents the best fit of Eq. 2 of Methods. Extracted IC_50_ values are listed in Table 1 with statistical information. Dotted lines reproduce the wild type for direct comparison in the same panel.

Parameters extracted from data shown in Figs 2, 3, and Supplementary Fig. S1. The changes in K_0.5_ or IC_50_ relative to wild type (Wt) are indicated as fold changes (×) shown by arrow (upward for increase and downward for decrease). Indicated by ± is s.d.; n, number of independent experiments performed for each assay.

### Apparent K^+^ affinity of Q925 mutants

Binding of K^+^ to the extracellular-facing configuration of sites I and II of the wild type E_2_P phosphoenzyme intermediate stimulates dephosphorylation (Fig. 1, reactions 5 and 6). Because all the Q925 mutants underwent Na^+^-activated phosphorylation, their affinity for K^+^ could be estimated by measuring the K^+^-induced depletion of the phosphoenzyme level as the enzyme was incubated with [γ-^32^P]ATP in the presence of 50 mM Na^+^ and various concentrations of K^+^ (Fig. 3 and Table 1). The wild type enzyme exhibited an IC_50_ value for K^+^ of ~0.05 mM, whether stably or transiently transfected. All mutants exhibited ≥400-fold reduced apparent affinity (increased IC_50_) for K^+^, in fact ≥1000-fold for the bulkiest substituents. Again, the result for Q925A was independent of the expression methodology used (Table 1). The conspicuous reduction of the apparent affinity for K^+^, seen even for the glycine and alanine mutations that increased intracellular Na^+^ affinity, indicates that Q925 is an essential residue for conferring sensitivity to K^+^. The insensitivity of the Q925 mutants toward K^+^ could be due to a direct effect on the intrinsic affinity for K^+^ or to a displacement of the E_1_P-E_2_P equilibrium toward the K^+^-insensitive E_1_P form (Fig. 1, reaction 4). We therefore analyzed the E_1_P-E_2_P distribution of the mutant phosphoenzyme (see Supplementary Fig. S1). All the mutants showed equal or larger E_2_P accumulation than wild type. Hence, the K^+^ insensitivity of the Q925 mutants must reflect a reduced intrinsic affinity of E_2_P for K^+^.

### ATPase activity of stably expressed Q925A and Q925G mutants

**Figure 4:**
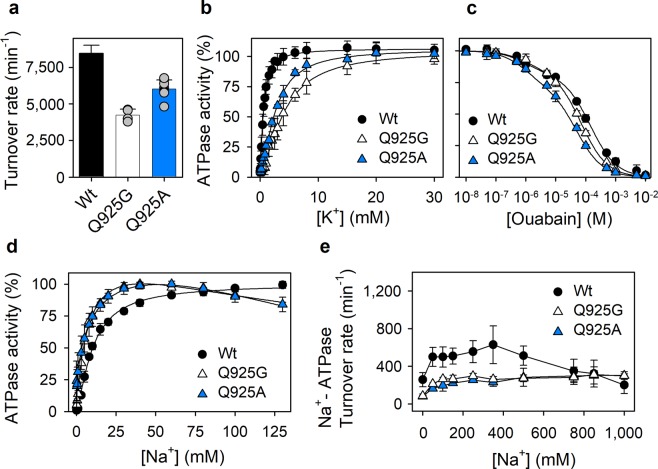
ATPase activity of stably expressed Q925A and Q925G mutants. The ATPase activity was determined at 37 °C in medium containing 30 mM histidine (pH 7.4), 1 mM EGTA, 3 mM MgCl_2_, and 3 mM ATP, together with the NaCl, KCl, and ouabain additions indicated below. Symbols and columns with error bars represent mean ± s.d. The lines show the best fits of the equations from Methods indicated below. Extracted parameters are listed in Table 2. Turnover rates were calculated as the ratio between the ATPase activity and the active site concentration (see Methods). (**a**) Medium additions: 130 mM NaCl, 20 mM KCl, and 10 μM ouabain. Individual data points are shown as grey circles. (**b**) Medium additions: 40 mM NaCl, 10 μM ouabain, and KCl as indicated. The extrapolated value corresponding to infinite K^+^ concentration was taken as 100%. Eq. (1) used for fitting. (**c**) Medium additions: 130 mM NaCl, 20 mM KCl, and ouabain as indicated. Eq. (3) used for fitting. (**d**) Medium additions: 20 mM KCl, 10 μM ouabain, and NaCl as indicated. The maximum was taken as 100%. K_0.5_ values for activation obtained by fitting Eq. 1. to the rising part of the Na^+^ dependence are listed in Table 2. The inhibition phase at high Na^+^ concentrations is caused by competition of Na^+^ with K^+^ at extracellular-facing sites due to low K^+^ affinity of the mutants. (**e**) Medium additions: 10 μM ouabain and NaCl as indicated, no KCl was present.

We characterized the ATPase activity of Q925A and Q925G, the mutants active under physiological conditions. At saturating Na^+^ and K^+^ concentrations for the wild type enzyme, the turnover rate of ATP hydrolysis was reduced in Q925A and Q925G relative to wild type (Fig. 4a and Table 2). Moreover, the K^+^ dependence of the ATPase activity of these mutants shown in Fig. 4b indicates that the apparent affinity for K^+^ was lower by ~5-fold for Q925A and ~8-fold for Q925G, relative to wild type (Table 2), thus further supporting the notion that mutation of Q925 affects K^+^ binding.

**Table 2:** Functional analysis by ATPase activity measurements.

	Turnover rate	ATPase activity		
		K_0.5_ (K^+^)	K_0.5_ (Na^+^)	IC_50_ (ouabain)
	min^−1^	μM	mM	μM
α1 Wt	8,474 ± 547 n = 11	587 ± 55 n = 8	10.4 ± 0.8 n = 6	141 ± 14 n = 4
Q925A	6,031 ± 615 (71% Wt) n = 8	3,128 ± 859 (5.3 ×↑) n = 3	3.3 ± 2.0 (3.1 ×↓) n = 5	49 ± 2 (2.9 ×↓) n = 4
Q925G	4,233 ± 408 (50% Wt) n = 4	4,571 ± 887 (7.8 ×↑) n = 6	4.3 ± 0.03 (2.4 ×↓) n = 3	70 ± 0.4 (2.0 ×↓) n = 2

Parameters extracted from data shown in Fig. 4a–d. The changes relative to wild type (Wt) are indicated as % of Wt (turnover rate) or fold change (×) relative to Wt shown by arrow (upward for increase and downward for decrease). Indicated by ± is s.d.; n, number of independent experiments performed for each assay.

The experiments described above were conducted in the presence of ouabain to inhibit the endogenous COS-1 cell Na^+^, K^+^-ATPase. Figure 4c shows the ouabain dependence of the total Na^+^, K^+^-ATPase activity of the membranes. Biphasic inhibition curves were obtained for both wild type and mutants. The phase with the smallest amplitude and IC_50_ around 1 μM corresponds to the endogenous COS-1 cell Na^+^, K^+^-ATPase. The prominent low-affinity phase starting around 10 μM corresponds to the exogenous expressed mutant and wild type rat enzymes. The Q925A and Q925G mutants exhibited 2- to 3-fold higher apparent ouabain affinity than the wild type (see Table 2 for the extracted IC_50_ values). Because of the well-known ouabain-K^+^ antagonism^19^, the increased apparent affinity for ouabain can be explained by the reduced affinity for K^+^ described above. The amplitude of the low-affinity phase corresponding to the relative contribution of the exogenous enzyme to the activity in the absence of ouabain was: 81%, 72%, and 85% for the wild type, Q925A, and Q925G, respectively. The contribution of the exogenous enzyme at 10 μM ouabain, where the ATPase activity measurements shown in Fig. 4a,b,d,e, were performed were 98%, 96%, and 98%, respectively (calculated from the IC_50_ values determined in Fig. 4c), thus providing confidence that the endogenous enzyme was efficiently silenced.

The Na^+^-dependence of Na^+^, K^+^-ATPase activity of Q925A and Q925G is shown in Fig. 4d. Similar to the phosphorylation studies reported in Fig. 2, the apparent affinity for Na^+^ activation of Na^+^, K^+^-ATPase activity reflects the interaction of Na^+^ at the intracellular-facing sites, although in the presence of K^+^. Again, a surprising increase of the apparent Na^+^ affinity at the intracellular-facing sites (2- to 3-fold) was revealed for Q925A and Q925G, relative to wild type.

To address the interaction with Na^+^ at the extracellular-facing sites, we measured the ATPase activity in the presence of Na^+^ without K^+^ (“Na^+^-ATPase activity”). Under these conditions Na^+^ not only activates the phosphorylation by binding at the three intracellular-facing sites, but also substitutes for K^+^ at sites I and II in the extracellular-facing configuration of the E_2_P form, thus promoting dephosphorylation, although at a 10–20-fold lower rate and with much lower affinity compared with K^+ ^^2,6,20^. These effects are seen as Na^+^ stimulation of the ATPase activity over a range of Na^+^ concentrations. However, because Na^+^ binding from the extracellular side of the membrane (at site III, see Discussion) also shifts the E_1_P to E_2_P transition back toward E_1_P (reaction 4, Fig. 1), an inhibition of the Na^+^-ATPase activity is seen at high Na^+^ concentrations for the wild type. In the Q925A and Q925G mutants, this inhibition was absent, thus revealing a defective Na^+^ binding at site III from the extracellular side. Furthermore, Fig. 4e shows that the maximal Na^+^-ATPase activity reached at high Na^+^ concentrations was markedly lower for the two mutants than for the wild type, suggesting a reduced efficacy of Na^+^ stimulation of dephosphorylation in these mutants. The latter effect is explained by interference with the Na^+^ interaction at sites I and II that promotes dephosphorylation in the absence of K^+^ (see Discussion).

### Electrophysiological evidence that Q925 mutations reduce external ion affinity

We carried out electrophysiological experiments for wild type and the Q925A, Q925L, and Q925E mutants expressed in *Xenopus* oocytes, to further evaluate the external ion-binding reactions. These three mutations were selected to represent a small and a large hydrophobic residue as well as a negatively charged residue isosteric with the glutamine of the wild type, and were introduced into a relatively ouabain-insensitive *Xenopus* α_1_ template (“RD-α_1_”, referred to as wild type for simplicity, see Methods). Mutants and wild type were co-expressed with *Xenopus* β_3_ in *Xenopus* oocytes. Two-electrode voltage clamp was used to measure the electric signals produced by these electrogenic Na^+^, K^+^-ATPases in a preparation with sided membranes.

**Figure 5:**
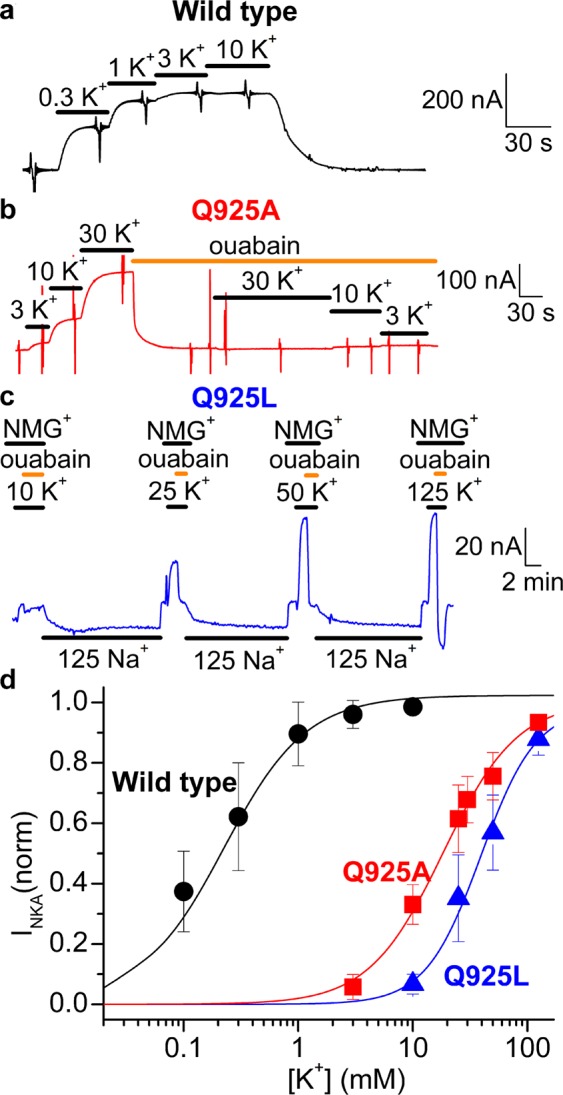
K^+^-concentration dependence of Na^+^, K^+^-ATPase currents in oocytes. (**a**–**c**) Representative current recordings at –50 mV (black, red, and blue traces). (**a**) Wild type-injected oocyte exposed to increasing concentrations of K^+^ (black lines) while bathed in NMG^+^ solution. K^+^-induced currents were fully saturated at 3 mM K^+^. (**b**) A similar experiment in an oocyte injected with Q925A. Here, much larger K^+^ concentrations (black lines) were required to activate Na^+^, K^+^-ATPase currents. Ouabain (10 mM, orange line) completely inhibited the K^+^-induced currents. (**c**) An oocyte injected with Q925L was exposed to 10 mM K^+^ (black line) followed by 10 mM ouabain (orange line), in NMG^+^ solution. Upon ouabain withdrawal in Na^+^ solution (black line) to recover from ATPase inhibition, the solution was switched to NMG^+^ where 25 mM K^+^ and subsequently ouabain were applied, uncovering Na^+^, K^+^-ATPase currents. The manoeuvres were repeated with 50 and 125 mM K^+^. (**d**) K^+^-concentration dependence of the current at –50 mV. Data points represent the mean ± s.d. Line plots are the Hill equations (Eq. 4. in Methods) fitted to the data from individual oocytes, with the K_0.5_ and statistics shown in Table 3. The global fit of the experiments used a single Hill coefficient shared among all the experiments for each mutant, with values n = 1.3 ± 0.1 for wild type, n = 1.3 ± 0.1 for Q925A and n = 1.8 ± 0.2 for Q925L (s.e.m. values as obtained from the fitting program). For wild type, the K^+^-induced currents obtained in the absence of ouabain are shown. For Q925A and Q925L, the ouabain-inhibited part of the currents are shown at all K^+^ concentrations, thereby eliminating contributions from K^+^ channels at high K^+^ concentrations.

**Figure 6:**
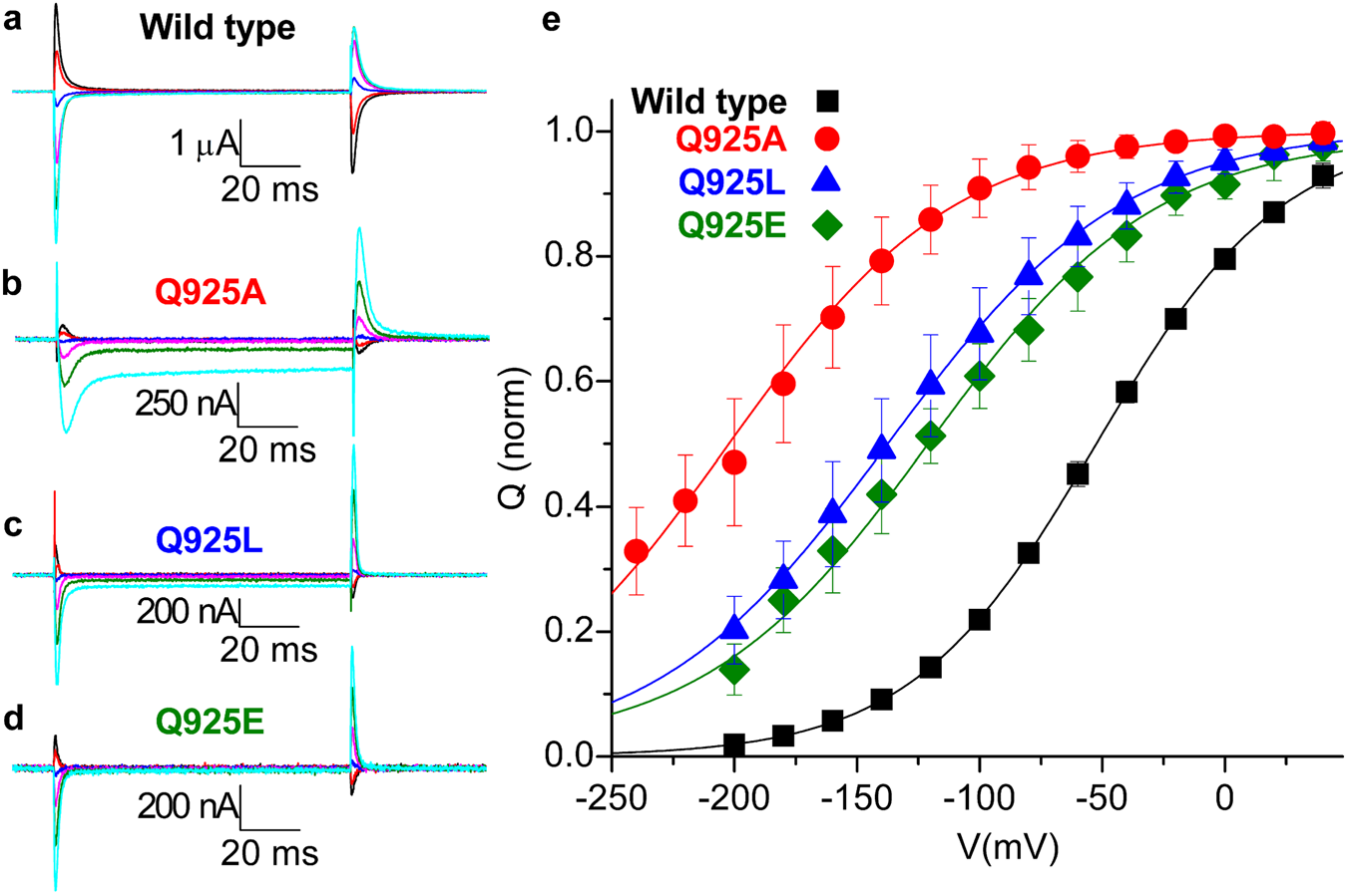
Transient charge movement in 125 mM Na^+^ without external K^+^. (**a**–**d**) Ouabain-sensitive currents elicited by voltage pulses from –50 mV to –180 mV (cyan), −140 mV (green), −100 mV (magenta), −60 mV (blue), −20 mV (red) and + 20 mV (black), in representative oocytes injected with wild type (**a**), Q925A (**b**), Q925L (**c**) and Q925E (**d**). (**e**) Normalized charge-voltage plots. Data points represent the mean ± s.d. Line plots are Boltzmann distributions (Eq. 5 in Methods) fitted to individual experiments with slope factors 38 mV for wild type, 46 mV for Q925A, 48 mV for Q925L, and 46 mV for Q925E. V_0.5_ values, and the corresponding fold changes in Na^+^ affinity calculated, are shown in Table 3 with further information on statistics.

The oocytes were loaded with Na^+^ to saturate intracellular-facing sites by suspending the cells in a Na^+^-loading solution, from which Na^+^ diffuses into the cells. The Na^+^-loading solution also contained 10 μM ouabain to irreversibly inhibit the oocyte’s endogenous pumps from the extracellular side (see Methods^21^). The K^+^ dependence of activation of outward current due to exchange of three Na^+^ for two K^+^ was then studied in the absence of external Na^+^, substituted with N-methyl-D-glucamine (NMG^+^), which does not interact with the binding sites, to avert ion competition for external sites (Fig. 5). Current traces from representative oocytes, held at a membrane potential of −50 mV, illustrate the effect of applying increasing K^+^ concentrations in different solutions (Fig. 5a–c). Submillimolar K^+^ concentrations induced robust outward currents in wild type-injected oocytes (Fig. 5a), an effect that saturated at ~3 mM, as previously shown^21^. In contrast, concentrations higher than 3 mM K^+^ were needed to activate significant current in Q925A (Fig. 5b). K^+^-induced currents at K^+^ concentrations up to 10 mM are mediated by Na^+^, K^+^-ATPases under our experimental conditions. However, contaminant K^+^-channel currents begin to appear above 10 mM K^+^ ^22^, making it necessary to determine the ouabain-sensitive fraction of the currents specifically associated with the Na^+^, K^+^-ATPase by inhibition with 10 mM ouabain. Ouabain-sensitive outward currents appeared at K^+^ concentrations above 10 mM in oocytes expressing Q925L (Fig. 5c), uncovering the enzymatic activity of this mutant unable to support COS-1 cell growth in the presence of ouabain under physiologic ion concentrations. Figure 5d shows the K^+^ dependence of the Na^+^, K^+^-ATPase currents for wild type, Q925A, and Q925L, normalized to the maximum from Hill fits to data from individual oocytes. The apparent affinities for K^+^ activation of the Na^+^, K^+^-ATPase currents extracted as half-maximal activating K^+^ concentrations from those fits were reduced 88- and 180-fold for Q925A and Q925L, respectively, relative to wild type (Table 3), in agreement with the reduced K^+^ affinity revealed by the phosphorylation data in Fig. 3. The maximal Na^+^, K^+^-ATPase current was wild type-like for Q925A and approximately 50% of wild type for Q925L, whereas the ouabain-sensitive current at 125 mM K^+^ in oocytes injected with Q925E was negligible (Table 3), although external Na^+^- and voltage-dependent transitions (see Fig. 6) demonstrate Q925E expression.

**Table 3:** Electrophysiological analysis of the mutants.

	Maximal current	K^+^ dependence of current		Q-V relationship		
		K_0.5_(K^+^)	n	V_0.5_	K_0.5_(Na^+^)	n
	nA	mM		mV	Fold change	
α1 Wt	334 ± 249	0.23 ± 0.19	24	–51.3 ± 6.1		23
Q925A	304 ± 106	20.2 ± 5.0 (88 ×↑)	12	–196 ± 17	96 ×↑	8
Q925L	149 ± 78	41.3 ± 11.6 (180 ×↑)	10	–137 ± 16	15 ×↑	9
Q925E	6.8 ± 2.9	n.d.	13	–121 ± 11	9 ×↑	9

Parameters extracted from data in Fig. 5 (K^+^ dependence of currents) and Fig. 6 (Q-V relationship). The Q-V relationship data for individual experiments were fitted by a Boltzmann distribution (see Methods), yielding centres, V_0.5_. The changes in K_0.5_ relative to wild type (Wt) are indicated as fold changes (×) shown by arrow (upward for increase and downward for decrease). Indicated by ± is s.d.; n, number of independent experiments performed for each assay; n.d., not determined because of too low current. The change in K_0.5_(Na^+^) relative to wild type was calculated from the shift of V_0.5_ assuming a shift of 22 mV for each 2-fold change of K_0.5_(Na^+^)^24^.

To estimate the mutational effects on external Na^+^ binding, we measured the transient charge movement that represents the voltage-dependent redistribution between [Na_3_]E_1_P and E_2_P intermediates^23^ (reaction 4 in Fig. 1), in the presence of 125 mM Na^+^ and absence of K^+^ (Fig. 6). The ouabain-sensitive transient currents elicited by application of voltage pulses from the holding potential of −50 mV in representative oocytes showed similar amplitudes in response to negative and positive voltages for wild type (Fig. 6a), but much larger currents for negative voltage pulses than for positive voltage pulses in Q925A (Fig. 6b), Q925L (Fig. 6c), or Q925E (Fig. 6d). This is an indication of reduced Na^+^ affinity, as more negative voltages are needed to push Na^+^ ions back into their binding sites, when the affinity for Na^+^ is reduced.

The integral of the transient currents observed when the voltage was returned to holding was plotted as a function of the voltage pulse to obtain the charge versus voltage (Q-V) relationship (Fig. 6e, normalized to account for different expression levels). The data for individual experiments were fitted by a Boltzmann distribution (see Methods). The Boltzmann’s centres (V_0.5_) for the mutants were shifted toward more negative voltages as compared with wild type. Such shifts reflect a reduced affinity for external Na^+^. A shift of 22–25 mV occurs for every 2-fold change of the external Na^+^ concentration^23,24^. Using a value of 22 mV^24^, the shifts in the V_0.5_ for Q925A, Q925L, and Q925E correspond to 96-fold, 15-fold, and 9-fold reductions, respectively, of the affinity for extracellular Na^+^ (Fig. 6e and Table 3). Thus, Q925A, one of the mutants with increased affinity for internal Na^+^ (Fig. 2), and with the smallest reduction of the affinity for external K^+^ (Figs 3 and 5d), exhibited the strongest reduction of the affinity for external Na^+^. This finding agrees with the lack of inhibition of Na^+^-ATPase activity at high Na^+^ concentrations seen for the Q925A mutant (Fig. 4e), which also reflects a defective Na^+^ binding from the extracellular side.

## Discussion

**Figure 7:**
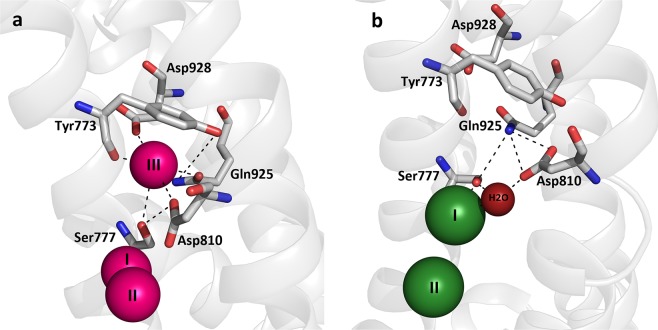
Ion-binding sites in Na^+^, K^+^-ATPase crystal structures with indication of Q925 (Gln925) and other relevant site-III residues. (**a**) Na^+^-bound [Na_3_]E_1_·AlF_4_^−^ ADP form (mimicking the transition state between Na_3_E_1_ and [Na_3_]E_1_P, see Fig. 1), Protein Data Bank code 3WGV, chain A (protomer B)^14^. Na^+^ ions are shown as pink spheres numbered I, II, and III according to standard nomenclature. (**b**) K^+^-bound [K_2_]E_2_·MgF_4_^2–^ form (mimicking the [K_2_]E_2_·P_i_ intermediate between K_2_E_2_P and [K_2_]E_2_), Protein Data Bank code 2ZXE^13^. K^+^ ions are shown as green spheres numbered I and II, and the associated water molecule is red. View along the plane of the membrane with the intracellular side up, for both structures. Selected residues are shown in stick representation coloured according to the elements (carbon, grey; oxygen, red; nitrogen, blue) and numbered according to the rat α_1_-isoform (Q925 of rat α_1_ corresponds to Q923 and Q930 in the crystallized pig α_1_ and shark rectal gland enzymes, respectively). Broken lines indicate potential hydrogen bonds or coordination bonds between ions and oxygen ligands of residue side chains.

Although the crystal structures of the Na^+^, K^+^-ATPase represent static snapshots of the interactions with the transported ions, they provide a solid starting point for discussion of the present data. The crystal structure with three bound Na^+^^14^, representing an intermediate between Na_3_E_1_ and [Na_3_]E_1_P, shows that Q925 is part of the Na^+^-selective site III (Fig. 7a), whereas the crystal structure of the K^+^-bound [K_2_]E_2_ form^12,13^ shows that Q925 is located too far away from the bound K^+^ to play a direct role as a K^+^ ligand (Fig. 7b). It was therefore surprising that the consequences of the Q925 mutations included severe effects on K^+^ affinity, whereas the Q925A and Q925G mutations in fact increased the affinity for Na^+^, binding from the intracellular side of the membrane. The reduced K^+^ affinity in the Q925 mutants was demonstrated in several assays: (1) The phosphoenzyme of all the Q925 mutants exhibited a strongly reduced sensitivity to K^+^ (Fig. 3), which was not caused indirectly by a shift of the E_1_P-E_2_P distribution in favour of the K^+^-insensitive E_1_P form (Supplementary Fig. S1). (2) The ouabain-sensitive K^+^-induced currents mediated by mutants Q925A and Q925L in the sided oocyte system showed a strongly reduced apparent affinity for K^+^ activation at the external sites compared with wild type. It is of note that mutant Q925L, which did not support cell growth in the mammalian cell culture, was able to generate Na^+^, K^+^-ATPase-mediated current across the oocyte membrane, when the extracellular K^+^ concentration was increased to unphysiologically high values (Fig. 5). (3) A reduced K^+^ affinity for activation of ATP hydrolysis was observed for the two active mutants Q925A and Q925G (Fig. 4b). 4) Q925A and Q925G furthermore exhibited an increased apparent affinity for ouabain in the presence of K^+^ (Fig. 4c), which reflects a reduced K^+^ affinity, because of the well-known ouabain-K^+^ antagonism^19^.

The reduced K^+^ affinity observed for the Q925 mutants seems to be due to disturbance of other residues and a water molecule involved in K^+^ binding at site I. Hence, the crystal structure in Fig. 7b shows that the amide nitrogen of Q925 participates in a hydrogen-bonding network that fixes both S777, which is directly involved in K^+^ binding at site I, and D810, which binds K^+^ via the water molecule. The Q925 mutations are expected to interfere with these bonds. Our data therefore attest to the strong importance of this bonding network, connecting the unoccupied site III with the K^+^ at site I in the K^+^-bound E_2_ state. The indirect interference with K^+^ binding at some distance from Q925 is apparent from the finding that the two mutations inserting the smallest side chains, Q925A and Q925G, were the least disruptive, whereas those inserting the most bulky substituents, Q925L, Q925I, and Q925Y, were the most disruptive. Note furthermore that Q925 could not even be semi-conservatively replaced with glutamate without a conspicuous, ~1000-fold reduction of the apparent K^+^ affinity, thus supporting a role of the amide nitrogen. The asparagine substituent caused a rather similar disruption of K^+^ binding (~400-fold reduction of the apparent K^+^ affinity, Table 1). Due to the shorter carbon chain length of the asparagine side chain compared with glutamine, the amide nitrogen of the asparagine is too far away from S777 and D810 to fix these residues by the correct hydrogen bonds (see Fig. 7b).

The role of Q925 in the binding of Na^+^ from the intracellular side of the membrane was addressed by measuring the apparent affinity for Na^+^ activation of phosphorylation, which involves E_1_ and E_1_P that encounter and occlude the Na^+^ ions entering the binding sites in their intracellular-facing configuration (Fig. 1). In the Na^+^-bound crystal structure (Fig. 7a)^14^, the side-chain oxygen atom of Q925 is within coordination distance of Na^+^ at site III. Therefore, the affinity for Na^+^ activation of phosphorylation was expected to be reduced by mutation of Q925. This was indeed born out for the mutants with the larger substituents. However, the increase of apparent Na^+^ affinity observed for the smallest substituents glycine and alanine in the phosphorylation (Fig. 2) and Na^+^, K^+^-ATPase assays (Fig. 4d) was unexpected, as it contradicts an essential role of the Q925 side chain as a Na^+^ ligand. Given the increased Na^+^ affinity seen for Q925A and Q925G, it seems likely that the reduced Na^+^ affinity observed for the other Q925 mutants is caused by formation of alternative hydrogen bonds (Q925N and Q925E), and/or by steric interference by the most bulky side chains (Q925L, Q925I, and Q925Y), rather than by the absence of an essential Na^+^-coordinating group. In the crystal structure, Q925 together with the other residues comprising Na^+^ site III, Y773, S777, T776, D810, and D928 (some being shown in Fig. 7) enclose a cavity containing the ion^14^. The bound Na^+^ ion may possess some freedom to move in the cavity, which does not fit the ion tightly, as suggested by the exclusive ability of site III to house a guanidinium ion in place of Na^+^^21,25–27^. A clue to how much freedom of movement the Na^+^ ion at site III has in the wild type enzyme comes from comparing the location of this Na^+^ ion in the two slightly different conformations of the α-subunit found in the crystal structure (in protomers A and B, see Fig. S10 of^14^). The Na^+^ ion is closest to Q925 in protomer B (distance 2.69 Å) and somewhat further away in protomer A (distance 3.03 Å). The two protomers in the crystal structure furthermore differ by the distance between the Na^+^ ion at site III and D928, another potential Na^+^-coordinating residue^28^ (4.20 Å for protomer A and 3.34 Å for protomer B). Mutations Q925A and Q925G, partly or totally removing the Q925 side chain, might facilitate movement of Na^+^ deeper into the site toward the negatively charged D928, which could lead to stabilization of the Na^+^ ion there, and thus to higher affinity.

In contrast to the increased affinity for intracellular Na^+^ observed for Q925A and Q925G, these mutants appeared strongly defective with respect to the binding of Na^+^ from the extracellular side, as indicated by the lack of inhibition of Na^+^-ATPase activity by Na^+^ at high concentration (Fig. 4e). For Q925A, the strongly reduced Na^+^ affinity on the extracellular side was documented by a conspicuous shift of the voltage dependence of transient charge movements toward negative voltage (Fig. 6), corresponding to as much as 96-fold reduction of the affinity for extracellular Na^+^. Therefore, Q925 is much more critical for Na^+^ binding from the extracellular side as compared with the intracellular side. On the other hand, Q925L and Q925E altered Na^+^ binding from both sides of the membrane, but on the extracellular side the disturbance was less than seen for Q925A. These differences must be a consequence of the conformational rearrangement occurring when the Na^+^ sites alter between intracellular-facing/occluded and extracellular-facing states. Various mutations of D928 and certain other residues in the C-terminal region that specifically reduce the affinity of Na^+^ site III, with little or no disturbance of sites I and II, have been found to interfere with the inhibition of Na^+^-ATPase activity by Na^+^ at high concentration^21,28–31^ in a way similar to mutations Q925A and Q925G (Fig. 4e). Hence, it appears that binding of the Na^+^ ion at site III is responsible for the inhibition of Na^+^-ATPase activity by Na^+^ at high concentration in the wild type, likely by shifting the E_1_P to E_2_P transition back toward E_1_P (reaction 4, Fig. 1), i.e. reversing the unloading of Na^+^ on the extracellular side^29^. It may therefore be concluded that mutations Q925A and Q925G disrupt the extracellular-facing configuration of site III. As Q925 is located toward the extracellular side relative to the bound Na^+^ at site III, it may be speculated that Q925 is a critical residue for normal external gating at that site. In such case, the larger reduction of the apparent affinity for extracellular Na^+^ seen for the substituents with the shortest side chains could be due to a widening of the gateway between site III and the extracellular side, resulting in a higher Na^+^ release rate and lower affinity of site III in the extracellular-facing configuration.

Our data furthermore indicate that Q925 plays an additional, important role in maintaining the structure of one or both of the two extracellular-facing sites (I and II) where K^+^ (or Na^+^ in Na^+^-ATPase activity) normally binds to activate the dephosphorylation of the wild type. Hence, we found a strong reduction by Q925 substitutions of the apparent K^+^ affinity and of the efficacy of Na^+^ to stimulate Na^+^-ATPase activity. A reduced efficacy of K^+^ (or Na^+^ in Na^+^-ATPase activity) to stimulate the dephosphorylation of E_2_P would explain the reduced turnover rate of ATP hydrolysis in Na^+^, K^+^-ATPase (or Na^+^-ATPase) assays of mutants Q925A and Q925G in the presence of ion concentrations that would be saturating for the wild type enzyme (Fig. 4a,e). In essence, the evidence points to site III being responsible for the inhibition of Na^+^-ATPase activity by reversal of the unloading of Na^+^ on the extracellular side, and sites I and II being responsible for pump activation through stimulation of dephosphorylation when either Na^+^ or K^+^ binds from the extracellular side, and Q925 is essential to both functions.

## Conclusion

Here we show that Q925 of the Na^+^-selective site III unexpectedly is a critical residue for K^+^ binding, which may be explained by the importance of the intricate hydrogen-bonding network between Q925 and site I, one of the two sites where K^+^ binds. Despite the location of the side-chain oxygen atom of Q925 within coordination distance of Na^+^ at site III in the Na^+^-bound crystal structure, replacement of Q925 with the small residues alanine and glycine increased the apparent affinity for Na^+^ binding from the intracellular side, a possible result of an increased freedom of movement of Na^+^ within site III in its intracellular-facing/occluded configuration, allowing Na^+^ to approach the negatively charged D928 in these mutants. By contrast, the Na^+^ affinity on the extracellular side was lowered upon replacement of Q925, particularly for the small substituents, which might be explained by a role of Q925 as a gating residue, whose movement is required to allow release of the Na^+^ ion toward the extracellular side. Hence, Q925 may be involved in Na^+^ interaction at site III in its extracellular-facing configuration, as well as indirectly in K^+^ and Na^+^ interaction at site I (and thereby also site II, as these sites interact). These results provide insight in the dynamics of Q925 in relation to the transformation of the ion-binding sites from intracellular- to extracellular-facing orientation during the ion translocation process.

## Methods

### Site-directed mutagenesis and expression for biochemical studies

Point mutations corresponding to amino acid substitutions Q925A/G/E/N/L/I/Y were introduced by PCR into the full-length cDNA encoding the naturally relatively ouabain-insensitive rat α_1_-isoform of Na^+^, K^+^-ATPase contained within the pMT2 expression vector^17^. The resulting constructs were full-length sequenced to verify the correct point mutation, both before transfection into COS-1 cells and after expanding single colonies into stable cell lines under ouabain-selection pressure, as previously described^5,17,18^. Cells were grown at 37 °C and 5% CO_2_ in DMEM medium (VWR Life Science) containing 1% penicillin, 1% non-essential amino acids, 10% fetal bovine serum. Transfection with the cDNA was carried out using the Ca^2+^-phosphate precipitation method^32^. Post transfection, the cells were treated with 10% glycerol to enhance DNA uptake. Ouabain selection of stable cell lines expressing the exogenous relatively ouabain-insensitive Na^+^, K^+^-ATPase was initiated by adding 1 μM ouabain to the DMEM medium three days post transfection. The ouabain concentration was later increased to 5 μM, ensuring a gradual reduction of the endogenous Na^+^, K^+^-ATPase activity that allows time for selection of the cells expressing the exogenous Na^+^, K^+^-ATPase at a high level. The stably transfected cells were grown under the ouabain-selection pressure until the expression level was fully optimized (in some cases 2 months or more), prior to isolation of the plasma membranes containing the expressed Na^+^, K^+^-ATPase. Survival of transfected cells during the cultivation in the presence of ouabain depends on the ability of the expressed relatively ouabain-insensitive exogenous Na^+^, K^+^-ATPase to transport Na^+^ and K^+^, because ouabain inhibits the endogenous, ouabain-sensitive Na^+^, K^+^-ATPase of the cells^5,17^. Generation of stable cell lines using the ouabain-selection method was only feasible for mutants Q925A and Q925G as well as the wild type. To express the transport-inactive mutants Q925E/N/L/I/Y, we used transient expression methodology, combining the Ca^2+^-phosphate precipitation method with siRNA to specifically silence the endogenous Na^+^, K^+^-ATPase. The siRNA used was designed to target the mRNA corresponding to part of the ouabain-binding site located in the extracellular loop between M1 and M2 of the endogenous COS-1 cell Na^+^, K^+^-ATPase^33^, where the sequence deviates from the rat α_1_ Na^+^, K^+^-ATPase. The siRNA sequence (5′-AA GCU GCU ACA GAA GAG GAA C-‘3) was determined by sequencing the COS-1 cell genomic DNA corresponding to exon 4. For transfection, 10 nM of the siRNA was added to the COS-1 cells together with the pMT2 vector containing the cDNA. Cells were grown and treated with glycerol, as described above for stable transfection, except for the absence of ouabain. The time from transfection of the COS-1 cells to harvest of the membranes in the transient transfection experiments was 72 hours. For the functional measurements on the isolated plasma membranes, ouabain was added to the media in order to inhibit the endogenous enzyme, both for stably and transiently expressed Na^+^, K^+^-ATPase. The expression level of the exogenous Na^+^, K^+^-ATPase obtained by the two expression methods can be compared by measuring the phosphorylation from [γ-^32^P]ATP under stoichiometric conditions (“active site concentration”) using the method described below in the presence of 100 μM ouabain to inhibit the endogenous Na^+^, K^+^-ATPase, yielding (mean ± s.d.) 19.1 ± 5.8 pmol/mg protein (n = 4) for stable transfection of the wild type, and 2.9 ± 0.5 pmol/mg protein (n = 4) for transient transfection of the wild type. In contrast, the background measured in mock transfected COS-1 cells was 0.05 ± 0.03 pmol/mg protein (n = 4), indicating that ouabain efficiently inhibited all endogenous enzyme. The transient expression level was 15% of the stable expression level, and close to 60-fold higher than the background. Hence, accurate functional measurements could be performed with the transiently expressed enzyme as well as the stably expressed enzyme, as demonstrated by the results obtained.

### Isolation of plasma membrane vesicles and ATPase activity measurements

The plasma membrane fractions containing the expressed wild type or mutants were isolated from the COS-1 cells by differential centrifugation, and made leaky with alamethicin to allow access of ATP and ions from both sides of the membrane^31^. In some cases alamethicin was compared with deoxycholate as permeabilizing agent. The kinetic parameters measured after deoxycholate treatment were indistinguishable from those obtained with alamethicin, except for a slightly lower maximal activity indicating inactivation. The ATPase activity of the transport-active Q925A and Q925G mutants as well as the wild type, expressed in stable cell lines, was determined on the leaky membranes using a colorimetric procedure^34^. The liberation of P_i_ from the added ATP was followed at 37 °C for 15–19 min (i.e. within the linear time range). The composition of the media is described in the figure legends. Except for the experiments where the ouabain concentration dependence was studied, the ouabain concentration was 10 μM to inhibit the endogenous COS-1 cell Na^+^, K^+^-ATPase. For background subtraction, similar measurements were carried out in the presence of 10 mM ouabain, inhibiting all Na^+^, K^+^-ATPase activity.

### Phosphorylation experiments

Phosphorylation studies with [γ-^32^P]ATP were performed on the leaky membranes according to previously established protocols^35,36^. The composition of the media is described in the figure legends. For examination of the stably expressed mutants, the medium contained 10 μM ouabain, whereas the ouabain concentration was increased to 100 μM for the transiently expressed mutants to make sure that the endogenous enzyme (making up a larger fraction of the total amount of Na^+^, K^+^-ATPase in this case) did not contribute to the results. The phosphorylation was quenched with ice-cold 1 M phosphoric acid, pH 2.4. The acid-precipitated ^32^P-labelled phosphoenzyme was washed by centrifugation, followed by SDS-polyacrylamide gel electrophoresis at pH 6.0, for approximately 1 hour at 15 °C to prevent any degradation of enzyme due to heat development. Visualization and quantification of the separated ^32^P-labelled phosphoenzyme were obtained by phosphor imaging using the Cyclone^®^/OptiQuant™ system from PerkinElmer Life Sciences.

The active site concentration was determined by phosphorylation from [γ-^32^P]ATP under stoichiometric conditions, i.e. as described for the Na^+^ dependence of phosphorylation (legend to Fig. 2) at a saturating Na^+^ concentration of 150 mM with 20 μg oligomycin/ml to prevent dephosphorylation, and ouabain to inhibit the endogenous enzyme. For calculation of the catalytic turnover rate, the ATPase activity determined as described above under ATPase activity measurements was related to the active site concentration^36^.

### Data analysis for biochemical experiments

Data points shown in figures are mean values with standard deviation (s.d.) indicated by error bars. All experiments were performed independently at least two times (three or more for most), and different preparations of isolated plasma membranes were used for each mutant and wild type. The SigmaPlot program (SPSS, Inc.) was used to fit relevant equations to data points using non-linear regression. The K_0.5_ and IC_50_ values were determined by fitting each independent set of data points, followed by calculation of the mean values and s.d. shown in Tables 1 and 2. The lines shown in the figures represent the best fit to all data points.

The Na^+^ dependence of phosphorylation and the Na^+^-, and K^+^-dependencies of Na^+^, K^+^-ATPase activity were fitted by a Hill function (Eq. 1):

1 $${\rm{A}}={{\rm{A}}}_{0}+{{\rm{A}}}_{{\rm{\max }}}\cdot (\frac{{[{\rm{L}}]}^{{\rm{n}}}}{{{\rm{K}}}_{0.5}^{{\rm{n}}}+{[{\rm{L}}]}^{{\rm{n}}}})$$

Here and in the equations below, A represents the actual phosphorylation level or ATPase activity at the given ligand (L) concentration. In Eq. 1, A_0_ is the basic activity in the absence of the ligand (zero for Na^+^ dependence), A_max_ is the extrapolated maximum value of the variable fraction corresponding to infinite ligand concentration (A_0_ + A_max_ = 100%). K_0.5_ is the ligand concentration giving half-maximum activation (reciprocal of apparent affinity), and n the Hill coefficient.

The K^+^ dependence of phosphorylation was fitted by representing the inhibitory ligand (i.e. K^+^) binding by a negative Hill function (Eq. 2):

2 $${\rm{A}}={{\rm{A}}}_{{\rm{\max }}}\cdot (1\mbox{--}\frac{{[{\rm{L}}]}^{{\rm{n}}}\,}{{{\rm{IC}}}_{50}^{{\rm{n}}}+{[{\rm{L}}]}^{{\rm{n}}}})+{{\rm{A}}}_{0}$$

A_max_ is the maximum value of the variable fraction corresponding to zero concentration of the inhibitory ligand. A_0_ is the phosphorylation corresponding to infinite ligand concentration, i.e. maximal inhibition. Here and below, IC_50_ denotes the ligand concentration giving half-maximum inhibition, n is the Hill coefficient.

The ouabain dependence of the ATPase activity was fitted by representing the inhibitory binding of ouabain (Ou) by two Hill functions corresponding to the endogenous COS-1 cell Na^+^, K^+^-ATPase with high ouabain affinity (set at 1 μM) and the exogenous enzyme with a lower variable affinity to be determined by the fitting (Eq. 3):

3 $${\rm{A}}={{\rm{A}}}_{{\rm{tot}}}-\frac{{{\rm{A}}}_{{\rm{endo}}}[{\rm{Ou}}]}{{{\rm{IC}}}_{50{\rm{endo}}}+[{\rm{Ou}}]}-\frac{{{\rm{A}}}_{{\rm{exo}}}[{\rm{Ou}}]}{{{\rm{IC}}}_{50{\rm{exo}}}+[{\rm{Ou}}]}$$

A is the actual ATPase activity represented by the sum of the ATPase activities of the endogenous and the exogenous enzymes. A_endo_ and A_exo_ are the maximal ATPase activities of the endogenous and exogenous enzymes, respectively, obtained in the absence of ouabain, and A_tot_ is their sum (100% activity).

### Electrophysiological experiments with oocytes

The mutations equivalent to Q925A/L/E of the rat α_1_ Na^+^, K^+^-ATPase were introduced by PCR into the Q120R-N131D mutant (“RD-α_1_”, referred to as wild type) of *Xenopus* α_1_ Na^+^, K^+^-ATPase (for consistency rat α_1_ numbering is used here, the *Xenopus* α_1_ Q932 corresponds to rat α_1_ Q925). Like the rat α_1_, the RD-α_1_ exhibits low affinity for ouabain, but can be inhibited by ouabain at high concentration (10 mM). The mMESSAGE mMACHINE™ SP6 Transcription Kit (Ambion) was used for cRNA *in vitro* transcription. Oocytes were prepared as described^37^ and injected with an equimolar mixture of *Xenopus* α_1_ and *Xenopus* β_3_ cRNA (50 ng α, 17 ng β) and kept for 3–6 days at 16 °C in a solution containing 100 mM NaCl, 2 mM KCl, 1.8 mM CaCl_2_, 1 mM MgCl_2_, and 5 mM HEPES (pH 7.4) supplemented with horse serum and Gibco™ Antibiotic-Antimycotic (Thermo Fisher Scientific). Before recording, oocytes were loaded with Na^+^ by a one hour incubation in a solution containing 90 mM NaOH, 20 mM tetraethyl ammonium-OH, 0.2 mM EGTA, and 40 mM HEPES, pH 7.2 with sulfamic acid. The Na^+^ loading solution was supplemented with 10 μM ouabain to inhibit the oocyte’s endogenous pumps (irreversibly during the duration of the experiments). Some wild type-injected oocytes were loaded with 150 mM HEPES, 20 mM tetraethyl ammonium-Cl, and 0.2 mM EGTA (pH 7.2, with NaOH). These two solutions produced indistinguishable results^38^. Two-electrode voltage clamp recordings were performed at room temperature (21–23 °C) as described^37,38^ using an OC-725C amplifier (Warner Instruments, Hamden, CT, USA) with a Digidata 1440 A/D board, a MiniDigi 1 A, and pCLAMP™ 10 software (Molecular Devices, Sunnyvale, CA, USA). Signals were filtered at 2 kHz and digitized at 10 kHz. Resistance of the 3 M KCl-filled microelectrodes was 0.5–1 MΩ. The experimental bath solutions were composed of 133 mM methane sulfonic acid, 5 mM Ba(OH)_2_, 1 mM Mg(OH)_2_, 0.5 mM Ca(OH)_2_, 10 mM HEPES, and 125 mM of either NMG^+^, Na^+^ or K^+^, pH 7.6. The final K^+^ concentration in dose response experiments was obtained by mixing the 125 mM NMG^+^ and 125 mM K^+^ solutions, to maintain osmolality. To inhibit all Na^+^, K^+^-ATPase currents, 10 mM ouabain was added.

Currents induced by K^+^ and sensitive to ouabain were fitted with a Hill equation (Eq. (4)):

4 $${\rm{I}}={{\rm{I}}}_{{\rm{\max }}}\cdot (\frac{{[{\rm{K}}]}^{{\rm{n}}}}{{{\rm{K}}}_{0.5}^{{\rm{n}}}+{[{\rm{K}}]}^{{\rm{n}}}})$$

I_max_ is the current activated at saturating K^+^, n is the Hill coefficient (ranging between 1 and 2) and K_0.5_ is the ion concentration producing half-maximal current activation.

Charge-voltage (Q-V) curves were fitted with a Boltzmann distribution as described^39^ (Eq. 5):

5 $${\rm{Q}}={{\rm{Q}}}_{{\rm{hyp}}}-\,(\frac{{{\rm{Q}}}_{{\rm{tot}}}\,}{1+{\exp }^{({{\rm{z}}}_{{\rm{q}}}{\rm{e}}({\rm{V}}-{{\rm{V}}}_{0.5})/{\rm{kT}})}})$$

Q_hyp_ is the charge moved with voltage pulses negative to the holding potential, Q_tot_ is the total charge moved, V_0.5_ is the centre of the Boltzmann distribution on the voltage axis, z_q_ is the apparent valence of a charge that traverses the whole electric field, e is the elementary charge, k is the Boltzmann constant, and T is the absolute temperature; kT/z_q_e is also known as the slope factor. Q-V curves from individual oocytes were normalized to eliminate variations caused by different expression levels by subtracting Q_hyp_ and dividing by Q_tot_. Data are presented as mean ± s.d.

## Supplementary information

Figure S1

## Data Availability

All source data for figures and tables in the present article are available from the corresponding authors on reasonable request.
