# Supplementary material for: Distinct effects of Q925 mutation on intracellular and extracellular Na+ and K+ binding to the Na+, K+-ATPase
Source: Sci Rep. 2019 Sep 16;9:13344. doi: 10.1038/s41598-019-50009-2 (PMC6746705; doi:10.1038/s41598-019-50009-2)
Supplement: Supplementary file 1 — Figure S1 [file 41598_2019_50009_MOESM1_ESM.pdf]

Supplementary Information

for the article

Distinct effects of Q925 mutation on intracellular and extracellular Na^+ and K^+ binding to the Na^+,K^+-ATPase

Hang N. Nielsen[1], Kerri Spontarelli[2], Rikke Holm[1], Jens Peter Andersen[1], Anja P. Einholm[1], Pablo Artigas[2]*, and Bente Vilsen[1]*

From the [1]Department of Biomedicine, Aarhus University, DK-8000 Aarhus C, Denmark, and the [2]Department of Cell Physiology and Molecular Biophysics, Center for Membrane Protein Research, Texas Tech University Health Sciences Center, Lubbock, TX 79430, USA

*Correspondence and requests for materials should be addressed to B.V. (e-mail: bv@biomed.au.dk) and P.A. (e-mail: pablo.artigas@ttuhsc.edu)

Supplementary Figure

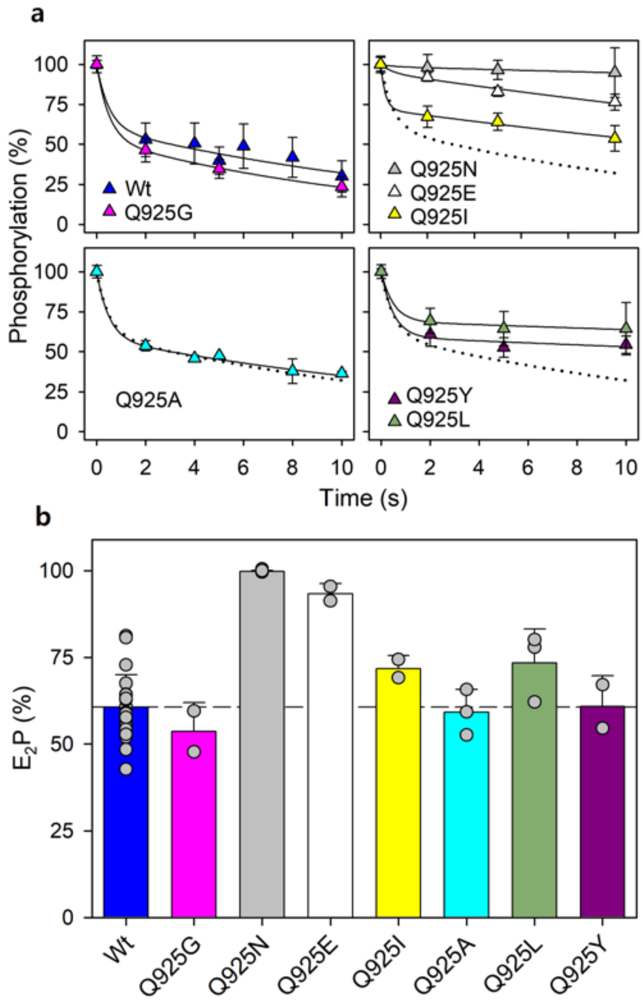

Figure S1. Distribution of the phosphoenzyme between E_1P and E_2P. (**a**) Phosphorylation was carried out by incubation of the leaky membranes with 2 µM [γ-^{32}P]ATP for 5 s at 0 °C in medium containing 20 mM Tris (pH 7.5), 50 mM NaCl, 3 mM $MgCl_2$, 1 mM EGTA, and ouabain to inhibit the endogenous enzyme (see Methods). To induce dephosphorylation, 2.5 mM ADP with 1 mM ATP (non-radioactive) was added followed by acid quenching at various time intervals. Symbols with error bars (seen only when larger than the size of the symbols) represent mean ± s.d. Each line shows the best fit of the bi-exponential decay function

$$EP = E_1P \cdot \exp^{(-k_1 t)} + E_2P \cdot \exp^{(-k_2 t)}$$

EP is the total amount of phosphoenzyme. E_1P and E_2P are the amounts of the two phosphoenzyme intermediates, k_1 and k_2 are the decay constants for the E_1P and E_2P phases, respectively. E_1P is ADP-sensitive, being able to donate the phosphoryl group back to ADP forming ATP in a rapid reaction, whereas E_2P dephosphorylates by hydrolysis – very slowly in the absence of K^+ – but is insensitive to ADP. Two clearly distinguishable phases of the dephosphorylation reaction are therefore seen upon addition of ADP to the phosphoenzyme: a rapid phase reflecting E_1P and a slow phase reflecting E_2P. Dotted lines reproduce the wild type for direct comparison in the same panel. (**b**) The relative amplitude of the slow phase corresponding to the E_2P fraction of the phosphoenzyme. All the individual data points extracted by applying the fitting procedure to each independent data set are shown as gray circles, and the mean values are indicated by the columns, with error bars indicating s.d. See also Table 1 of the main article. None of the mutants showed less accumulation of E_2P than the wild type. In fact, the E_2P fractions of the two mutants Q925E and Q925N were higher than that of the wild type.
